# Novel Machine Learning Analysis Algorithm of DNA Methylation Patterns Identifies Cerebral Palsy with Concurrent Epilepsy

**DOI:** 10.21203/rs.3.rs-4560364/v1

**Published:** 2024-06-19

**Authors:** Jonathan Hicks, Karyn Robinson, Stephanie Lee, Adam Marsh, Robert Akins

**Affiliations:** 1 Bioinformatics and Computational Biology, University of Delaware; 2 Nemours Children’s Health, Wilmington DE, USA

**Keywords:** Spastic Cerebral Palsy, Epilepsy, Diagnostics, Machine Learning, Support Vector Machines, Linear Discriminant Analysis

## Abstract

**Background:**

Spastic cerebral palsy, the most common pediatric-onset disabling condition with an estimated prevalence of 0.2% in children, is a complex condition characterized by stiff movement, muscle contractures, and abnormal gait that can diminish quality of life. Spastic CP accounts for approximately 83% of all CP cases and frequently co-occurs with other complex conditions, like epilepsy. An estimated 42% of spastic CP cases have co-occurring epilepsy. Unfortunately, CP is often difficult to diagnose. Although most children with CP are born with it or acquire it immediately after birth, many are not identified until after 19 months of age with CP diagnosis often not confirmed until 5 years of age. New bioinformatic approaches to identify CP earlier are needed. Recent studies indicate that altered DNA methylation patterns associated with CP may have diagnostic value. The potential confounding effects of co-occurrent epilepsy on these patterns are not known. We evaluated machine learning classification of CP patients with or without co-occurring epilepsy.

**Results:**

Whole blood samples were collected from 30 study participants diagnosed with epilepsy (n=4), spastic CP (n=10), both (n=8), or neither (n=8). A novel Support-Vector-Machine learning algorithm was developed to identify methylation loci that have ability to classify CP from controls in the presence or absence of epilepsy. This algorithm was also employed to measure classification ability of identified methylation loci. After preprocessing of data, isolation of important methylation loci was performed in a binary comparison between CP and controls, as well as in a 4-way scheme, encapsulating epilepsy diagnoses. The classification ability was similarly assessed. CP Classification performance was evaluated with and without inclusion of epilepsy as a feature. Median F1 scores were 0.67 in 4-class comparison, and 1.0 in the binary classification, outperforming Linear-Discriminant-Analysis (0.57 and 0.86, respectively).

**Conclusion:**

This novel algorithm was able to classify study participants with spastic CP and/or epilepsy from controls with significant performance. The algorithm shows promise for rapid identification in methylation data of diagnostic methylation loci. In this model, Support Vector Machines outperformed Linear Discriminant Analysis in classification. In the evaluation of epigenetics-based diagnostics for CP, epilepsy may not be a significant confounding factor.

## Background

Cerebral palsy (CP) is a major cause of lifelong physical disability. According to surveillance programs in the United States of America, CP affects about 1 in 345 children[1]. There are multiple types of CP. Approximately 83% of children with CP have the spastic type[1], which is characterized by high muscle tone, slow development of motor skills, and stiff movement[2]. For spastic CP patients, the quality of life can be severely affected by difficulty with movement, and independent functioning is often challenging. Although most children with CP are born with it, diagnosis is challenging and often delayed with the median age at diagnosis estimated at 19 months[3].

Confidence in a CP diagnosis is also low early in life until the condition is confirmed, typically at about 5 years of age[4]. With such a delay in clear diagnosis, an essential target window for early intervention is often missed. Successful diagnosis at a younger age would allow for earlier interventional strategies such as corrective surgery, therapy, or environmental enrichment that may greatly improve quality of life[5, 6, 7]. There is a great need in this population for a reduction in diagnostic age and an increase in diagnostic confidence. Bioinformatics and multi-omics approaches have shown promise in diagnosis and need to be explored further to understand diagnostic potential[8]. Despite the prevalence and often profound effects of CP, the fundamental cellular and molecular mechanisms contributing to CP remain largely uncharacterized. An improved understanding of the fundamental pathways associated with CP is needed to improve diagnosis and provide new avenues for advanced clinical management.

It is well recognized that significant inflammatory, infectious, or physiologically stressful events during late gestation or the perinatal period are contributing factors in the genesis of CP[9][10]. Acute inflammation, pre-term birth, and maternal infection are also associated with alterations to the methylome[11], and it has been proposed that the onset of CP may be associated with alterations in DNA methylation patterns[8]. Shifts in DNA methylation can be sustained long-term and passed to daughter cells during mitosis, propagating potentially distinguishing DNA methylation patterns that could potentially serve as early biomarkers for disease[12][13]. Recent work suggests that DNA methylation pattern differences are associated with spastic CP[8][14]. Since DNA methylation differences may include elements that arise at disease onset and that are relatively stable over time, advanced methylome analyses could contribute to improved diagnostics.

Work on CP methylomics to date has largely focused on binary comparisons between affected individuals and controls. The potential confounding effects of concurrent diagnoses in identifying DNA methylation differences in spastic CP, however, have not been evaluated. Concurrent diagnoses are common in CP; in particular, 42% of patients with spastic CP also have a diagnosis of epilepsy[1]. This high concurrence of diagnoses far exceeds the general prevalence of epilepsy in the population, which has been estimated at less than one percent. This large difference between expected and observed co-occurrence rates suggests that the two disease states are fundamentally related.

In this study, we examined relationships in the DNA methylation patterns in peripheral blood cells from study participants in four pediatric cohorts based on diagnosis: (i) spastic CP requiring surgery for scoliosis but without epilepsy; (ii) epilepsy without CP, (iii) both spastic CP requiring surgery for scoliosis and epilepsy; (iv) idiopathic scoliosis requiring surgery without epilepsy or CP (control group). We evaluated the ability of machine-learning models to classify CP and epilepsy based on DNA methylation patterns. The eventual aim of this project is develop a diagnostic assay with high information yield and accuracy in a younger demographic. In this study, success is defined by an F1 score in classification of greater than 0.8.

## Methods

Thirty children were enrolled in this study in four cohorts: control (n = 8), spastic CP without epilepsy (n = 10), epilepsy without CP (n = 4), and spastic CP with epilepsy (n = 8) after IRB-approved informed consent / assent. Descriptive statistics of the cohort can be found in supplemental information Section 1.

### Methylation Assay

Whole blood samples were collected and processed with Gentra Puregene DNA isolation kits. The isolated DNA was analyzed using Illumina MethylationEpic 850k kits at the Children’s Hospital of Philadelphia following the manufacturer’s recommendations, to identify the degree of methylation at CpG sites on the genome. DNA quality was assessed by agarose gel electrophoresis before conversion. Quality of the samples was assured post-bisulfite conversion with methylation-specific PCR of hMLH1.

### Probe Filtering and Preprocessing

The methylation data were filtered to remove potentially problematic probes, including probes that were (i) not statistically significantly detected by Illumina, (ii) SNPs, or (iii) sex-linked. The methylation intensity data were then log-odds transformed to M-values to addresses issues of heteroscedasticity[15]. ComBat batch-correction was performed to remove any batch effect from multiple runs of the methylation assay[16]. Both before and after batch-correction, any probes with infinite M-values were removed to avoid indeterminate calculations.

### Identification of Informative Probes

With a stratified bootstrap analysis (Table 1), 22 training and 8 test samples were selected at random 10,000 times(stratified approximately proportionally to group size). For each iteration of the bootstrap, an FDR-corrected F-test was performed on all probes, given the training set. This analysis was conducted using minfi’s dmpFinder function[17]. It is worth noting that in the 4-class case, the F statistic was calculated from the differences between the cerebral palsy(n=10) and control(n=8) groups. Only two groups can be used in an F test, so the samples with epilepsy are not used in the F test. In the 2-sample case, the presence or absence of epilepsy was not considered. Only diagnosis(n=18) or lack(n=12) of cerebral palsy was used in the F-test. In the binary analysis, issues arising from the low sample size of epilepsy were minimized, and the training-testing split was set to 23–7 instead of 22–8 to better approximate an 80–20 split. If the FDR-corrected p-value for a test was less than 0.001, the probe was reserved for further identification. If there were fewer than 2 probes that met such a criteria, that particular iteration of the bootstrap was removed as ML techniques require more than one independent variable. Once a set of high performing probes was identified, a radial SVM and maximum likelihood estimator LDA were built upon the subset, and was used to evaluate performance. For each probe in an iteration, a score was then generated using EQ1.

EQ1: Score generation equation for a probe

$$\text{Score}=F_{1,\text{weighted}}*-{log}_{10}\left(P_{\text{Uncorrected}}\right)$$

In this equation, the P value is used in place of an FDR-corrected P value, as it more directly represents statistical discrimination between groups. *F*_1_ scores are calculated as the harmonic mean of precision and recall, reflecting overall performance of a model, where accuracy would not be a good metric due to class imbalance.

This process was iterated 10,000 times and scores for each probe used were averaged across all iterations. If a probe was not used in a certain iteration (as its FDR P-value was greater than 0.001), it was ignored and given a score of 0. After all iterations, average scores were assumed to follow a multi-modal distribution as not every probe identified with a low p value was expected to be significant in discrimination. Otsu’s method was used to extract the higher-scoring probes within each distribution[18]. Because the number of high-performing probes was relatively low, thus potentially violating the assumptions of Otsu’s method, dip tests for multimodality were performed on each new distribution of the extracted probes[19]. If multimodality was detected, the entire process was reiterated using only the subset of higher-performing probes identified by the last iteration of Otsu’s method. This process was repeated until the dip test did not detect multimodality at *α* = 0.05.

Linearly dependent variables provide no novel information to the model. For this reason, after the subset of highest-performing probes was identified, each variable was correlated. For any pairing of probes with a variance-inflation-factor greater than five, only the highest-performing probe was kept. This process removed linear dependence and generated a more parsimonious set of probes for analysis.

### Validation of Probe-Selection Scheme

To validate the ability to identify significant CpGs in classification, a synthetic dataset was used with 25 control cases and 25 positive cases. In this synthetic dataset 1 million probes were generated using a uniform distribution of percent methylation load (bounded from 0 to 100). In addition, 5 probes were generated with high discriminability between groups, 10 with medium discriminability, and 15 with low discriminability. High discriminability is defined as a difference in methylation-load between groups between 25 and 40%, as well as a standard deviation of the groups between 6 and 8%. A medium discriminability probe is defined as a probe with a mean difference between 15 and 30% methylation load, with standard deviation between 8 and 10%. A low discriminability probe is generated with a mean difference between 10 and 20% and standard deviation between 10 and 12%.

These generated set of probes were first filtered down to meet assumptions of a biomarker. Three criteria were applied to each probe for both controls and positives. This accounted for six total tests for each probe. A probe was kept in this filtration step if the criteria was met 4 times with that probe. 1) the 95% confidence interval of a probe of either group encompassed less than a 40% methylation difference. 2) the mean methylation of a group was less than 10 percent or greater than 90 percent. 3)the standard deviation of a probe in either group was less than 40 percent methylation. If the criteria were met 4 times for any probe, the probe was kept ONLY if there was also a 10% mean difference in methylation between the control and positive.

### ML Performance Analysis of Identified Probes

Probe sets identified by each analysis condition were implemented into another bootstrapped analysis using the same ML technique (e.g. a probe set built to identify CP from control using SVM was implemented into an SVM model). Over 10,000 iterations, a stratified train-test split was made following Table 1. For each iteration, a corresponding ML model was created and assessed by its performance on the testing set. The median one-vs-rest F1 is reported among all 10,000 iterations and was used to compare performance.

### Sensitivity Analysis of ML Models

Synthetic validation samples were generated to assess model sensitivity. Synthetic validation was used in lieu of traditional validation due to sample size limitations. For each identified CpG in an ML model, 30 samples were generated for each group(that is, 60 samples total for binary analysis and 120 samples total for 4-way analysis), following a uniform distribution bounded by a multiple of the standard deviation(see EQ2). The sample CpG was allowed to vary between 1 and 10 standard deviations from the mean of the M value, and model classification ability was plotted for each multiple of the standard deviation.

EQ2: Equation to generate synthetic samples. A Uniform distribution is created for each CpG bound by the mean of that CpG’s m-value for its group plus or minus some multiple of the standard deviation of the CpG’s M-value for its group.

$$X\sim U\left(\mu_{CpG,\text{group}}-A\sigma_{CpG,\text{group}},\mu_{CpG,\text{group}}+A\sigma_{CpG,\text{group}}\right)$$

### Validation on External Datasets

A dataset containing mononuclear leukocytes from term infants(5 controls, 5 positive for histologic chorioamnionitis) was obtained from the Gene Expression Omnibus. The methylation data were converted to M-values and filtered down to contain only the 6 identified CpGs in separating CP from control. The data were then subjected to principal components analysis and observed for data separability.

The linear separability of the data guided a linear-kernel SVM to be trained on the 6 CpGs with the intent of classifying histologic chorioamnionitis from control. The model was bootstrapped with 10,000 iterations and was proportionally stratified into an 80–20 split. F1 scores of the testing samples were reported over all 10,000 iterations.

## Results

### Study Participants

The descriptive statistics of the four cohorts included in this study are listed below in Table 2 with additional information and preliminary statistical analyses on study participants listed in Supplemental Information Section 1. Diagnostic (ICD10) codes used for inclusion and exclusion criteria are included in Supplemental Information Section 2. There were no statistically significant differences found between groups in age (two-sided t-test, p = NS) or sex(t-test of proportions, p = NS). There was a statistically significant difference in Gross Motor Function Classification Score(GMFCS) between those with CP and those with both CP and epilepsy, reflecting the increased co-occurrence of epilepsy in children (two-sided t-test p = 0.015) who are more severely affected by CP[20]

### Methylation Assay

DNA was isolated from whole blood samples using Gentra Puregene DNA isolation kits (Minneapolis, Minnesota), per the manufacturer’s specifications. DNA was then processed, analyzed, and validated at the Center for Applied Genomics at the Children’s Hospital of Philadelphia using the Illumina Methylation Epic 850K panel kit. This technique uses bisulfite conversion of unmethylated cytosines to uracil via oxidative deamination and site-specific hybridization to detect discrepancies between converted and unconverted DNA where the presence of a methyl group on the 5’ ribose carbon blocks the bisulfite deamination reaction[21]. DNA integrity was assessed by agarose gel electrophoresis before conversion and quality control was performed post-bisulfite conversion using a methylation-specific PCR of hMLH1. After quality control, raw data were returned to Nemours for all analyses.

### Probe Filtering and Preprocessing

Data preprocessing was performed to reduce external sources of variation and error. Probes corresponding to common single nucleotide polymorphisms were excluded to avoid potential misreads due to variants. Probes were subsequently excluded if the signal variance was not significantly different from background variance of the probe at *α* = 0.05. Probes were then further refined to remove sex-related features to omit the influence of X chromosome suppression in females. For identification of sex-related probes and SNPs, the human genome assembly hg19 was used[22]. The remaining methylation data were converted from raw red and green fluorescence signals to M-values, which are log-odds transformations of the methylation intensity signals that are less heteroscedastic than other metrics[15]. In cases of complete methylation or complete nonmethylation (i.e., M-values of ± infinity) the probe was removed (n=21). Lastly, the ComBat algorithm was used to remove batch-related effects in the data[16]. In total, 806,777 probes remained after preliminary filtering. See Supplemental Information Section 3 for probe retention at each step.

### Identification of Informative Probes

Performing a stratified bootstrap with 10,000 iterations, probes were given a score based on their P-value of differential methylation(F-test, no FDR correction) and their ability to classify on testing samples following EQ. 1 in the methods.

Otsu’s method was performed to threshold filter low-scoring probes, as scores were found to be multi-modal based on the distributions of performance (Supplemental Information Section 4)[18]. If a dip test identified further multi-modality at *α* = 0.05, the process (including bootstrapping) was repeated with the higher scoring part of the distribution identified by Otsu’s method. This process was repeated until a dip test failed to detect multi-modality at *α* = 0.05. See Table 3 for final counts of probes identified by each of the four ML approaches.

### Validation of Probe-Selection Scheme

Synthetic data sets were generated with defined levels of group-specific discrimination (signal) and individual subject variance (noise). These sets had a small number of informative features (n=30) with three levels of discriminability defined by the mean and variance of a signal site among synthetic subjects. These signal sites were then embedded in a noisy background of random features (n=10^6^).

Synthetic data that were pre-filtered to meet certain assumptions were analyzed on the ML pipeline. Pre-filtration resulted in 48,391 potential biomarkers. After this prefiltration, The ML pipeline was used to evaluate identification ability of probes with intentional difference. The total detection is described in Table 4.

### ML Performance Analysis of Identified Probes

After identification of a best-performing subset of probes that was approximately unimodal, a 10,000-iteration bootstrap was again performed for each analysis condition on the identified subset of probes from that analysis. In this instance, P values and FDR-Corrected P values were not calculated. Each probe set generated in Table 3 was used to generate an ML model. Performance was measured as a one-vs-rest (o-v-r) weighted F1 score, and overall performance is reported as the median score among all 10,000 bootstrapped iterations An F1 score of 0.5 is considered random performance, and scores range from 0 to 1, with 1 being perfect classification. The objective of this study is to have median F1 scores of greater than 0.8. Results can be seen in Table 5. Though the median performance in the case of binary SVM is 1, it is worth noting that not all instances of the model yielded an F1 of 1. In this instance, 54.11 percent of the predictions had an F1 of 1, where the remaining 45.89 percent had performance lower than 1.

**Figure 1:**
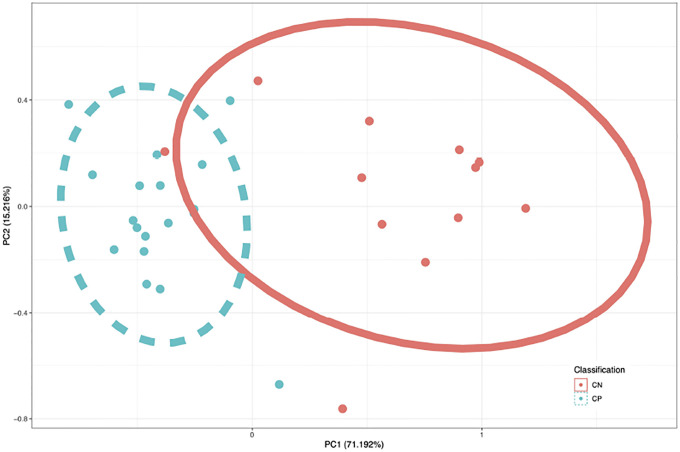
Principal Components Analysis performed on M-values identified only by binary comparison(n=6). The CpGs identified by either binary scheme are used in a PCA plot, where the first principal component contains 71.192% of the total variance of the 6 probes. The data are largely linearly separable.

**Figure 2:**
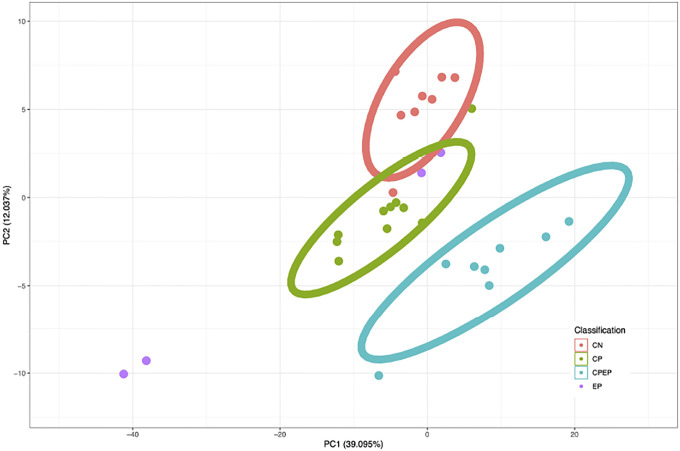
Principal components analysis performed on the M-values of the probes identified by any probe identification scheme (n=1,579). The CpGs identified by either 4-way scheme are used in a PCA plot, where 60.064% of the total variance is captured in principal component 1. The groups are largely separated. NOTE: Epilepsy M-values were not considered in the generation of this PCA plot.

For visualization of data separability, a PCA plot was generated using the combined set of probes identified across all ML techniques. This PCA plot demonstrated discriminative ability of the identified probes in the binary comparison and is shown in Figure 1. Probes identified to classify all four groups were used to generate a PCA plot showing discriminative ability of the more parsimonious CpG set. This is shown in Figure 2.The M-values for the epilepsy group were not considered in the generation of the PCA plot. The sample size of the epilepsy group was too low to gain reasonable confidence about group parameters, and so the epilepsy group was transformed and plotted onto a PCA generated from the other three groups. Though this PCA does not perfectly portray variance of the 30 samples, this PCA does in fact represent a valid transformation of the data that is visible to SVM and LDA models, and which shows the separability of data without giving undue consideration to an under-represented group in the data.

### Sensitivity Analysis of ML Models

**Figure 3:**
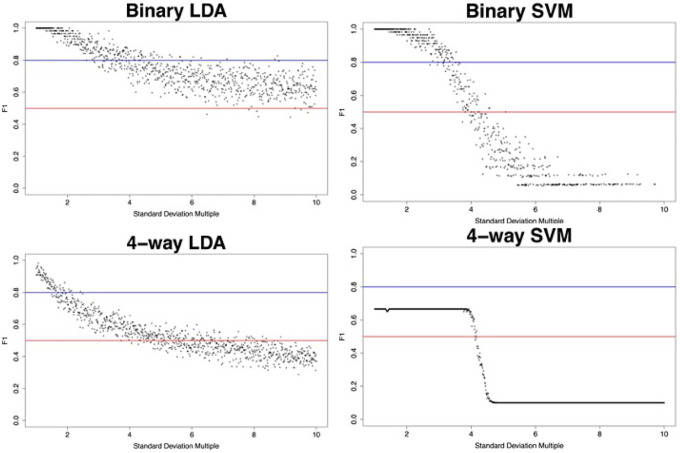
Synthetic data scoring subject to variance scaling from 1x to 10x. The red horizontal line at *F*1 = 0.5 indicates expected performance of a theoretical null model. The blue horizontal line at *F*1 = 0.8 indicates performance targeted by this study. Model performance outperforms a null model through at least 4 standard deviations of data in all models.

Sensitivity analyses of the ML models were conducted using from synthetic data with controlled signal:noise properties. Random uniform distributions of data were created, bounded by the mean M-value of a CpG in its group plus or minus a multiple of the standard deviation of the data to add variance in a quantified fashion. The ML models were tasked with classification of the generated synthetic data and F1 score was assessed as a measure of classification performance. See Figure 3. Performance of any model is able to outperform a theoretical null model for at least 3 standard deviations from the mean.A null model is defined by an algorithm not trained on any features, which predicts randomly. Because SVM models have a radial kernel, an increase in variance of the model will disproportionately increase samples outside of the decision boundary, leading to performance worse than a null model. In the case of LDA, with a linear decision boundary, an increase in variance will proportionally increase samples on both sides of a decision boundary, tending towards a null model. This explains the different tail behavior of the sensitivity analyses.

### Validation on External Datasets

**Figure 4:**
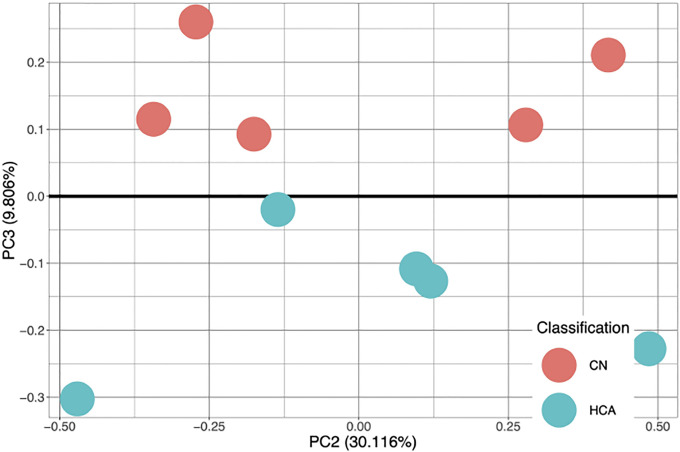
Principal Components Analysis performed on M values identified only by binary comparison(n=6) on an external dataset of mononuclear leukocytes identified as controls or chorioamnionitis samples. The third principal component is the primary separating axis. The two groups separate cleanly at the third principal axis

A methylation dataset of human cord blood mono-nuclear leukocytes from term neonates was obtained from the Gene Expression Omnibus[23] from the lab of Dr. Zubair Aghai[24]. This dataset includes 5 control samples and 5 samples positive for histological chorioamnionitis, a large risk factor for CP[25]. This dataset was filtered to only contain the 6 CpGs identified by the binary models. A PCA plot was generated to view separability and is shown in Figure 4.

An SVM model was trained on the filtered data using a linear kernel. In a similar manner, the data were bootstrapped with 10,000 iterations of an 80–20 split and F1 scores were reported. Median F1 score of classification was 0.667 and mean F1 score of classification was 0.810.

## Discussion

Identification of spastic CP is often delayed until after critical target windows for treatment have been missed. Current assessments for spastic CP are subjective, and there is a great need for an objective test performed in conjunction with routine examination in newborns and infants. A reduction in diagnostic age from an objective test that is part of routine care could drastically improve quality of life for afflicted individuals. Results from our LDA and SVM approaches indicate that methylome analysis readily identifies spastic CP cases versus non-CP controls regardless of the presence of epilepsy and may be reasonably capable of classifying epilepsy among those cohorts.

It is noteworthy that the 4-way SVM algorithm that included the epilepsy diagnosis identified a relatively large number of probes. This result may indicate that epilepsy is not inherently tied to methylation such that additional probes were required to achieve the F1 performance that we see. This may also explain the moderate performance of methylation pattern analysis in classification of epilepsy. For both ML techniques, the one-vs-rest weighted F1 scores were better than random. An F1 value greater than 0.5 is indicative of better-than-random performance and 1 is indicative of perfect performance. With median scores of 0.667 and 1.000 in the 4-way and 2-way classifications respectively, the performance of these models within study conditions is generally considered to be significant to profound. Binary classification was above the defined success criteria of 0.8. Important to the objective of the study, the presence or absence of an epilepsy diagnosis did not affect a model’s ability to classify CP.

Although the number of CpG sites was large when considering SVM diagnosis of epilepsy, all these loci were not needed for the classification of cerebral palsy. The set of loci created with the intention of classifying only CP is parsimonious but still effective as a diagnostic tool. We note that the set of probes used to classify CP in future studies and clinical applications may need to be larger to increase performance, but even a large increase in probes would result in relatively mi-nor increase in overall test complexity. Measurement of 100 CpG loci is not meaningfully more complicated than measurement of 10 CpG loci. Ongoing work seeks to validate the CpG set for CP classification and the feature set’s efficacy at CP classification at a lower age.

The principal component analysis shown in Figure 1 clearly demonstrate the separability of CP from non-CP. Given that the principal component analysis in Figure 2 demonstrated that most groups cluster tightly and separately, there is reason to believe that the identified probe set is efficient at discerning groups in this population. This is further supported by F1 scores of the model. Sensitivity analysis of these models using synthetic data (Figure 3) showed that even though parameters of the population are not known with high confidence, the model can be expected to be robust out beyond 3 standard deviations. Thus, the pipeline can be expected to perform well with larger and novel data streams. The SVM-models of sensitivity analysis converge towards F1 = 0 whereas the LDA models converge towards F1 = 0.5. It is believed that as variance increases, the LDA model, operating on a linear discriminant, will tend towards a theoretical null model as data falls on either side of the discriminant. SVM on the other hand, operating on a radial kernel, will not tend towards randomness as variance increases, as an increase of variance will increase sample count on one side of the decision boundary disproportionately.

The histologic chorioamnionitis samples in Figure 4 showed that the 6 identified CpGs are robust at identification of CP-related diseases and that the models work on external data (including samples of a much younger age). An F1 score of 0.667 indicated better than random prediction and indicates these CpGs should be able to effectively work in CP diagnostics in a younger population. Principal components of the dataset shows linear separability of data, so it is expected that with ideal hyperparameter selection, it may be possible to increase classification scores towards F1=1. Testing performance for all schemes, while good, might be further improved with the incorporation of other healthcare data. It has been shown in previous literature that Apgar scores, neonatal seizures, and head size can be different in children with epilepsy[26]. It is possible that with more healthcare-based data, such as social determinants of health, gestational age at birth, Apgar score, etc., a better model could be created to classify both cerebral palsy and epilepsy. Future studies are needed to assess the ability to classify with higher performance.

### Limitations

In this study, access to samples was a limiting factor, and so the group sizes are small and unequal. This is particularly noteworthy in the epilepsy group, with a sample size of four. With such a small sample size, confidence is significantly lowered, creating an under-representation of the epilepsy group in classification. For other groups, sample size was relatively small, but the confidence ellipsoid was still tightly clustered with minimal overlaps.

Another limiting factor Identified within this study is the difference in GMFCS scores between those with CP and epilepsy and those with CP. For the control group and for the epilepsy group, GMFCS was not measured, but there is a discrepancy between the other two groups at p = 0.015. This discrepancy is another difficulty faced with low sample size. The available study participant population did not allow for matching of this criterion, and it has been demonstrated in previous literature that concurrent epilepsy is associated with higher GMFCS score[20]. Given the ability of the models created to classify CP in the presence or absence of epilepsy, this is likely not a significant limitation to the study.

## Conclusion

With high F1 scores, support vector machines provide a high level of performance that may prove valuable in the assessment and classification of individuals with CP and epilepsy from those without those conditions based on DNA methylation patterns. Support vector machines should be considered in place of linear discriminant analysis for research in methylome classification assays of these diseases. The model evaluation metrics support the potential diagnostic capability of DNA methylation analyses to augment current diagnostic approaches for spastic CP and epilepsy. Sensitivity analyses show that the model is robust to variation in sample space and maintains performance even with an increase in variance.

## Supplementary Material

1

## Figures and Tables

**Table 1:** Sampling of the data to preserve group representation. NOTE: In the instance of 4-way comparison, the stratification for the epilepsy group reflects 2 for training and 2 for testing. In binary comparison, stratification reflects 3 training and 1 testing. This is done to preserve epilepsy samples in the testing group

Group	Total Sample Size	Training Sample Size	Testing Sample Size
Control	8	6	2
Spastic CP	10	8	2
Epilepsy	4	2	2
Spastic CP + Epilepsy	8	6	2
Total	30	22	8

**Table 2:** Descriptive statistics of each group. GMFCS is not measured for the control and the epilepsy group because GMFCS is not a part of standard care for these participants.

Group	Sample size	Age (*Mean* ± *SD*)	GMFCS Score (*Mean* ± *SD*)
Control	8	13.5 ± 2.1	Not Measured
Cerebral Palsy w/o Epilepsy	10	13.0 ± 5.0	2.300 ± 1.160
Epilepsy w/o Cerebral Palsy	4	12.6 ± 3.8	Not Measured
Cerebral Palsy and Epilepsy	8	11.6 ± 4.2	3.875 ± 1.246

**Table 3:** Counts of identified probes based on analysis performed. A 4-way comparison identified 10–1,575 probes whereas the binary comparison identified 5–6 probes.

ML Type	Comparison Type	Number of probes detected
LDA	Binary(All with CP vs all without CP)	5
SVM	Binary(All with CP vs all without CP)	6
LDA	All classes(4-way)	10
SVM	All classes(4-way)	1,575

**Table 4:** Probe identification ability in synthetic data. 100% of high-discriminability probes were detected, 80% of medium-discriminability probes were detected, 6% of low-discriminability probes were detected, and no false positives were generated.

Probe Discriminability	Probes in Group	Probes included by ML algorithm
High	5	5
Medium	10	8
Low	15	1
None(False Positive)	106	0

**Table 5:** Median performance of each ML analysis. A score can range from 0 to 1, with 0.5 indicating random performance. A higher score is better. Score distributions were non-normal, so median score is reported.

ML Type	Comparison Type	Median O-V-R *F*_1_	Probes Detected
LDA	Binary*	0.857	5
SVM	Binary*	1.000	6
LDA	All classes(4-way)	0.567	10
SVM	All classes(4-way)	0.667	1,575

* All with CP vs all without CP

## Data Availability

The datasets generated for this study will be stored in DbGaP pending approval for publication. The dataset referenced in this study can be found in the NCBI Gene Expression Omnibus with accession GSE153668 https://www.ncbi.nlm.nih.gov/geo/query/acc.cgi?acc=GSE153668

## List of Abbreviations

CP: spastic cerebral palsy
LDA: linear discriminant analysis
SVM: support vector machines
ML: machine learning
SNP: single nucleotide polymorphism
PCA: principal component analysis
GMFCS: gross motor function classification system
IRB: Institutional Review Board
