# Supplementary material for: Novel Machine Learning of DNA Methylation Patterns to Diagnose Complex Disease: Identification of Cerebral Palsy with Concurrent Epilepsy
Source: Res Sq. 2024 Sep 18:rs.3.rs-4560364. Originally published 2024 Jun 19. Preprint. [Version 2] doi: 10.21203/rs.3.rs-4560364/v2 (PMC11213172; doi:10.21203/rs.3.rs-4560364/v2)
Supplement: Supplement 1 [file NIHPPrs4560364v2-supplement-1.pdf]

Supplemental Information

Section 1

Study participant demographics and overview of statistical analyses for inter-group comparison. No statistically significant differences were found between the groups, with the exception of gross motor function classification score(GMFCS) between CP and CPEP groups.

Table S1: Participant Demographics

**Sample**	**Sex**	**Age**	**Cohort**	**GMFCS**
CN01	F	12	Control	N/A
CN02	M	15	Control	N/A
CN03	F	12	Control	N/A
CN04	F	14	Control	N/A
CN05	M	17	Control	N/A
CN06	M	14	Control	N/A
CN07	M	14	Control	N/A
CN08	F	10	Control	N/A
EP01	F	11	EP	N/A
EP02	F	18	EP	N/A
EP03	F	7	EP	N/A
EP04	M	16	EP	N/A
CP01	M	16	CP	1
CP02	F	17	CP	3
CP03	F	12	CP	2
CP04	F	5	CP	4
CP05	M	13	CP	3
CP06	M	9	CP	4
CP07	F	16	CP	2
CP08	M	10	CP	2
CP09	F	12	CP	1
CP10	F	16	CP	1
CPEP01	M	8	CPEP	2
CPEP02	F	14	CPEP	5
CPEP03	F	5	CPEP	4
CPEP04	M	13	CPEP	5
CPEP05	F	19	CPEP	5
CPEP06	M	13	CPEP	4
CPEP07	M	10	CPEP	4
CPEP08	F	11	CPEP	2

Table S2: Comparison of Cohorts

**Test**	**Group Comparison**	**Tested Value**	**p-Value**
Tukey's HSD	CP vs Control	Age	0.956
Tukey's HSD	CP and Epilepsy vs Control	Age	0.748
Tukey's HSD	Epilepsy vs Control	Age	0.996
Tukey's HSD	CP and Epilepsy vs CP	Age	0.946
Tukey's HSD	Epilepsy vs CP	Age	0.998
Tukey's HSD	Epilepsy vs CP and Epilepsy	Age	0.931
T Test	CP vs CP and Epilepsy	GMFCS	**0.015**

Section 2

Inclusion criteria for cohorts (ICD10 codes by group). The list of inclusion and exclusion ICD codes for each classification group. In addition to the inclusion/exclusion criteria, each participant must have had a blood draw as an additional inclusionary criterion.

Table S3: Inclusion and Exclusion ICD codes

**Group**	**Criteria Type**	**ICD10 Code**	**Description**
Control	Inclusionary	M41.0 - M41.9<br>Q67.5<br>M96.3	Scoliosis Conditions<br>Congenital Scoliosis<br>Post-laminectomy kyphosis
Control	Exclusionary	M41.4<br>M00.X - M99.X<br>G80.X<br>Q90.X - Q99.X<br>E88.X	Neuromuscular scoliosis<br>Musculoskeletal diseases<br>Any CP-related condition<br>Chromosomal abnormalities<br>Metabolic disorders
Cerebral Palsy	Inclusionary	G80.0-G80.2	Spastic cerebral palsy
Cerebral Palsy	Exclusionary	G80.3<br>G80.4<br>Q90.X - Q99.X<br>E88.X	Athetoid cerebral palsy<br>Ataxic cerebral palsy<br>Chromosomal abnormalities<br>Metabolic disorders
Epilepsy	Inclusionary	G40.0XX<br>G40.1XX<br><br>G40.2XX	Localization-related idiopathic epilepsy<br>Localization-related symptomatic epilepsy with simple partial seizures<br>Localization-related symptomatic epilepsy with complex partial seizures
Epilepsy	Exclusionary	G40.5XX<br><br>G40.8XX<br>G40.AXX	Epileptic seizures not related to external causes<br>Other epilepsy<br>Absence epileptic syndrome

Section 3

Probe-reduction by filtering. This filtering step is performed to assure that confounding variance is minimized, by removal of known high-variance probes. After filtering, 806,777 probes remained for subsequent analysis.

Table S4:

**Procedure**	**Remaining Probes**
Start	1,051,943
Remove single-nucleotide polymorphisms (SNPs)	835,424
Remove low detection-quality probes	825,328
Remove sex-chromosome probes	806,798
Removes probes with infinite M-values	806,777

Section 4

Distributions of probe performance after first iteration of bootstrapping. The scale of the graphs indicate a high number of low-performing probes and a small number of very-high-performing probes.

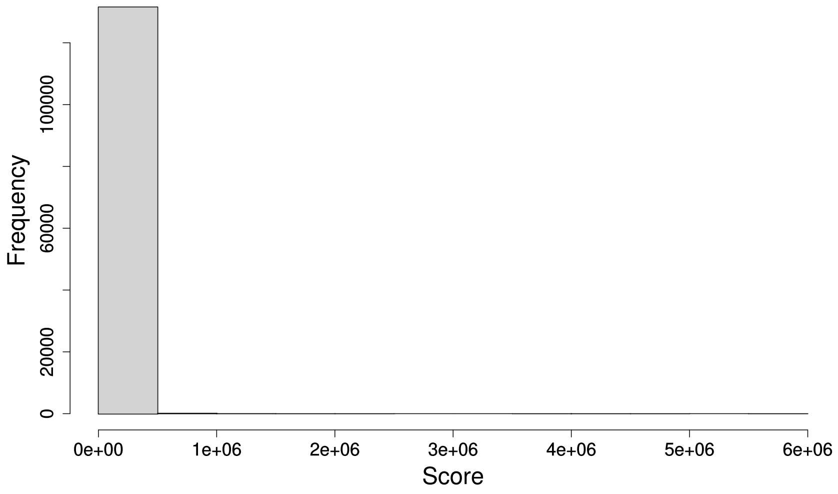

Figure S1: 4-way SVM scores

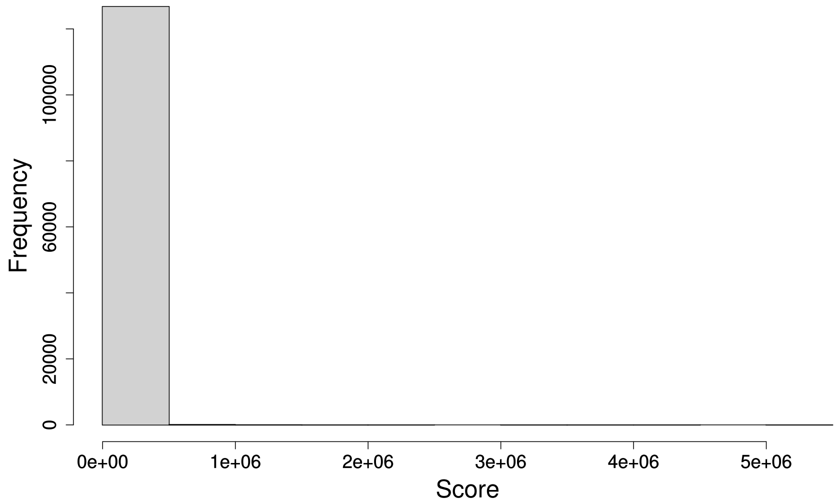

Figure S2: 4-way LDA scores

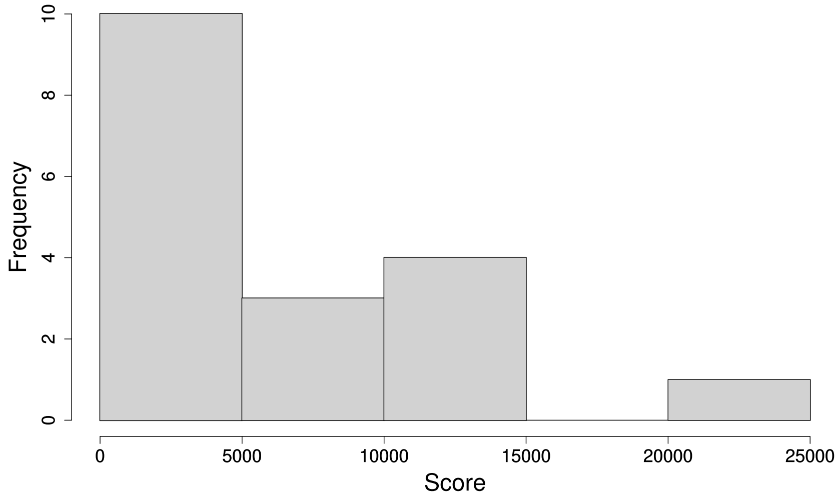

Figure S3: Binary SVM scores

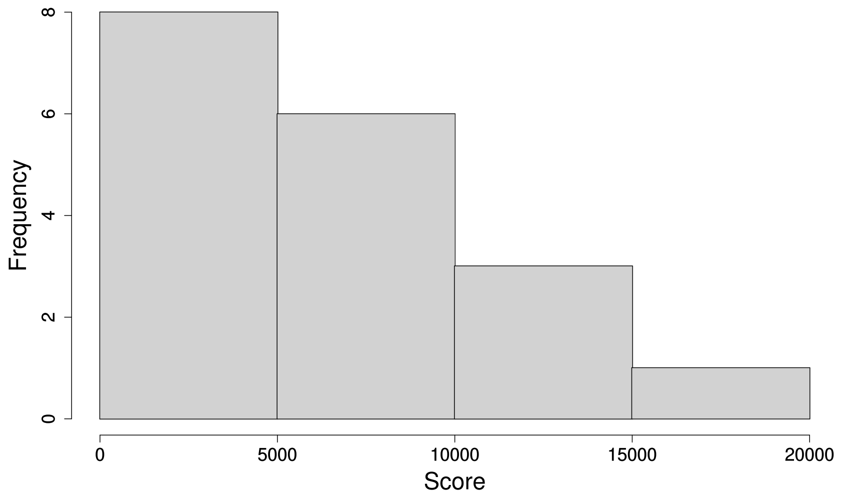

Figure S4: Binary LDA scores

Section 5

Flow Charts Illustrating Algorithm

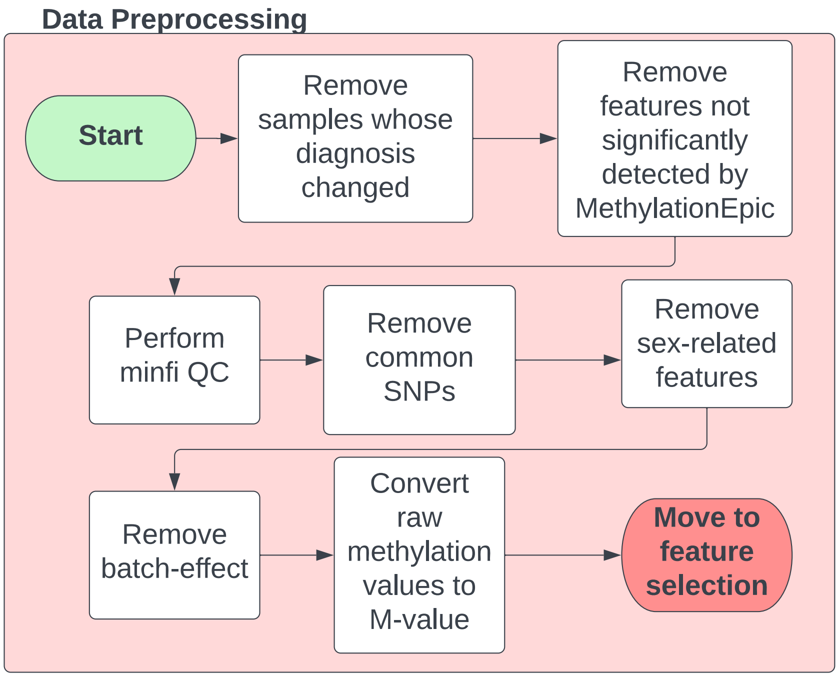

Figure S5: Data preprocessing algorithm. Data are filtered and processed from raw methylation data into M values. These values are used in the Important Feature Selection step.

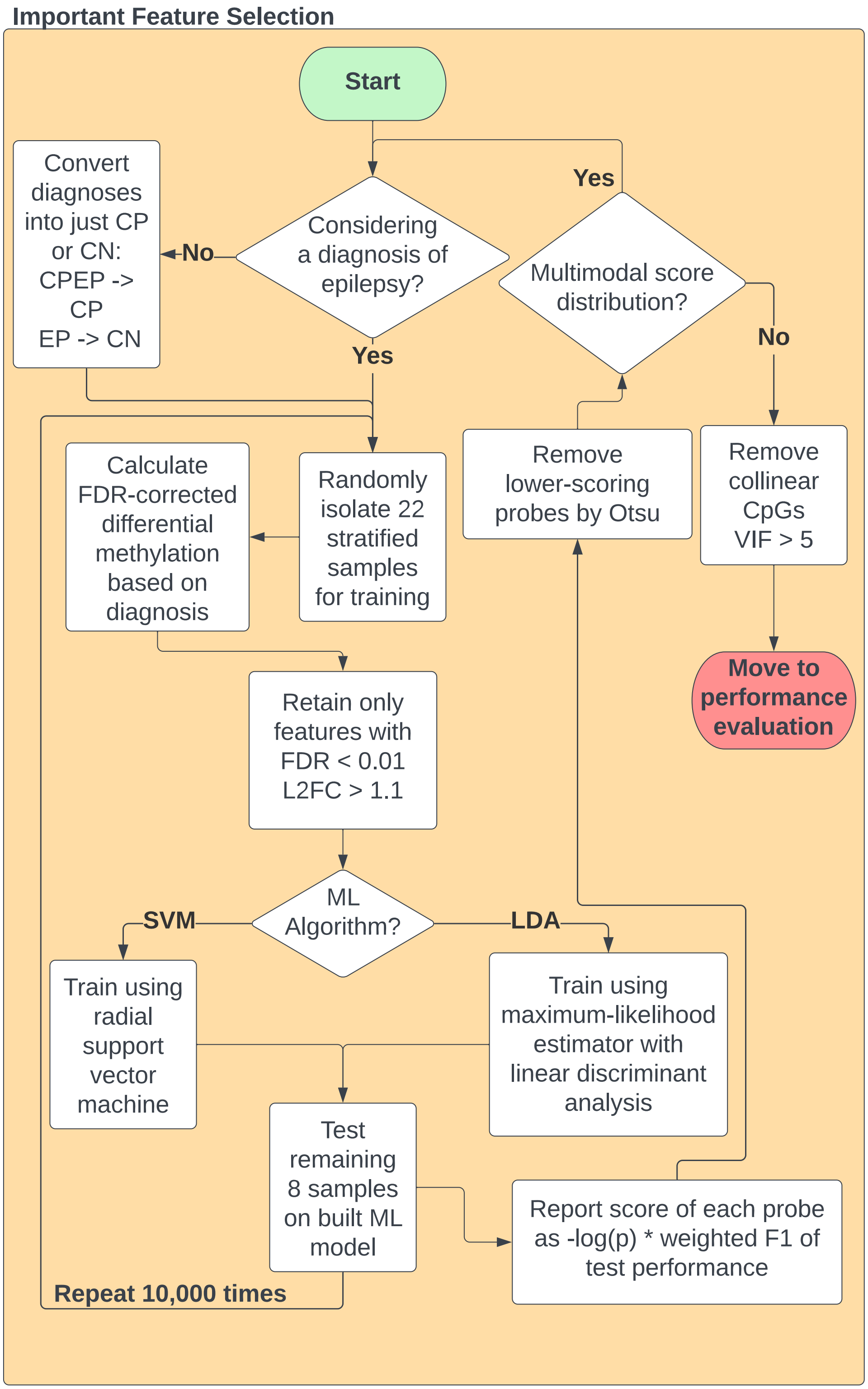

Figure S6: Important Feature Selection to identify informative CpG probes. CpG loci are identified that have a statistical difference between groups and high capacity to classify groups. Probes are run through the workflow until only high-performing probes remain. These probes move forward to evaluation.

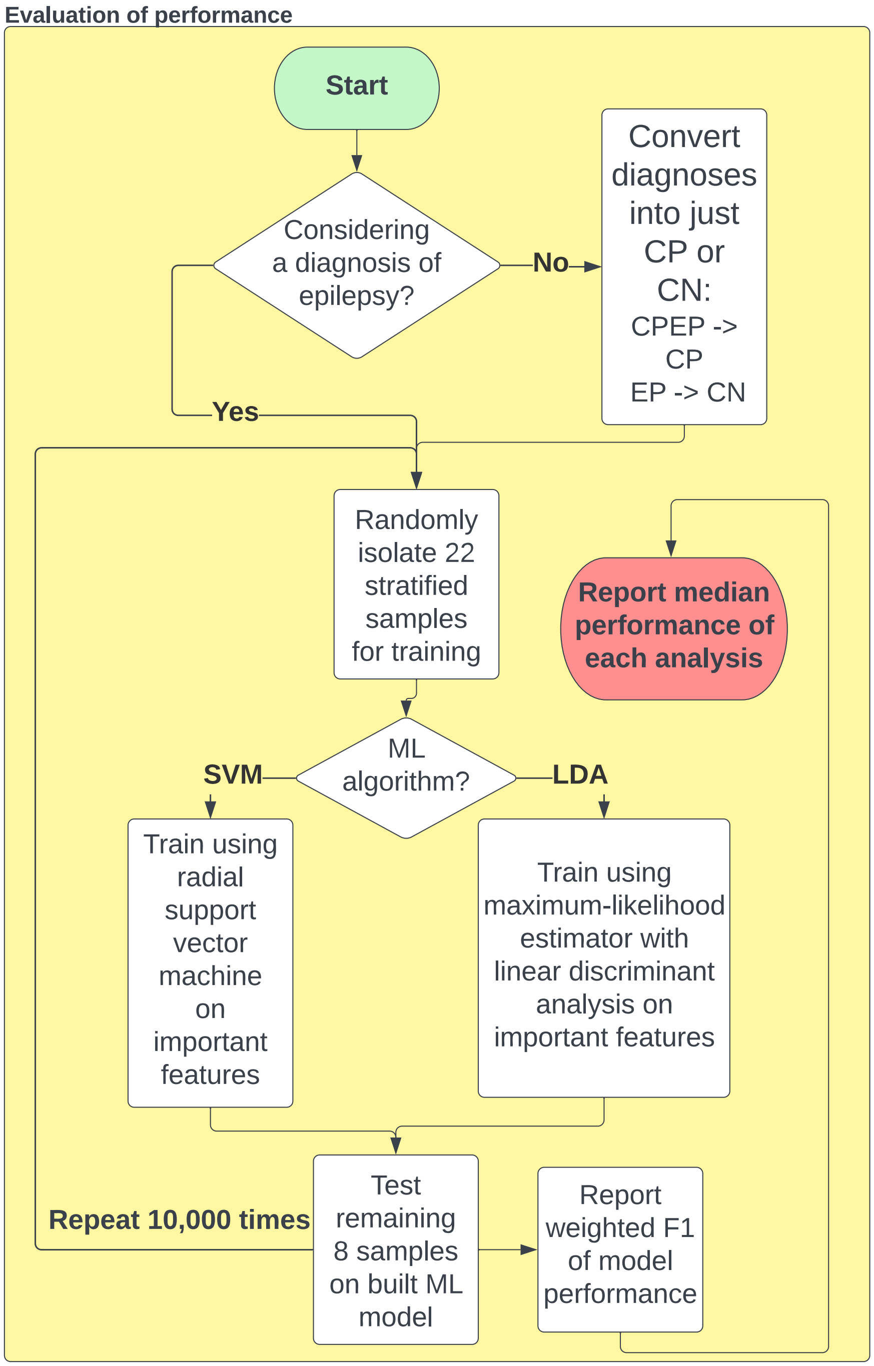

Figure S7: Evaluation of performance in a classification algorithm. In the yellow block, CpG loci that have been identified are used to assess diagnostic capability in the samples.
