# Supplementary material for: Novel Machine Learning Analysis Algorithm of DNA Methylation Patterns Identifies Cerebral Palsy with Concurrent Epilepsy
Source: Res Sq. 2024 Jun 19:rs.3.rs-4560364. Preprint. [Version 1] doi: 10.21203/rs.3.rs-4560364/v1 (PMC11213172; doi:10.21203/rs.3.rs-4560364/v1)
Supplement: Supplementary file 1 [file NIHPPrs4560364V1-supplement-1.pdf]

Supplemental Information

Section 1

Overview of study participants, and statistical analyses for inter-group comparison. No statistically significant difference is present in the groups, with the exception of GMFCS between CP and CPEP groups.

**Sample**	**Sex**	**Age**	**Diagnosis**	**GMFCS**
CN01	F	12	Control	N/A
CN02	M	15	Control	N/A
CN03	F	12	Control	N/A
CN04	F	14	Control	N/A
CN05	M	17	Control	N/A
CN06	M	14	Control	N/A
CN07	M	14	Control	N/A
CN08	F	10	Control	N/A
EP01	F	11	EP	N/A
EP02	F	18	EP	N/A
EP03	F	7	EP	N/A
EP04	M	16	EP	N/A
CP01	M	16	CP	1
CP02	F	17	CP	3
CP03	F	12	CP	2
CP04	F	5	CP	4
CP05	M	13	CP	3
CP06	M	9	CP	4
CP07	F	16	CP	2
CP08	M	10	CP	2
CP09	F	12	CP	1
CP10	F	16	CP	1
CPEP01	M	8	CPEP	2
CPEP02	F	14	CPEP	5
CPEP03	F	5	CPEP	4
CPEP04	M	13	CPEP	5
CPEP05	F	19	CPEP	5
CPEP06	M	13	CPEP	4
CPEP07	M	10	CPEP	4
CPEP08	F	11	CPEP	2

Test	Group Comparison	Tested Value	P Value
Tukey's HSD	CP vs Control	Age	0.956
Tukey's HSD	CP and Epilepsy vs Control	Age	0.748
Tukey's HSD	Epilepsy vs Control	Age	0.996
Tukey's HSD	CP and Epilepsy vs CP	Age	0.946
Tukey's HSD	EP vs CP	Age	0.998
Tukey's HSD	EP-CP and Epilepsy	Age	0.931
T Test	CP vs CP and Epilepsy	GMFCS	**0.015**

Section 2

Inclusion criteria for cohorts (ICD10 codes by group). The list of inclusion and exclusion ICD codes for each classification group. In addition to the inclusion/exclusion criteria, each participant must have had a blood draw as an additional inclusionary criterion.

Group	ICD10 Code	Description	Criteria Type
Control	M41.0 - M41.9<br>Q67.5<br>M96.3	Range of Scoliosis Conditions<br>Congenital Scoliosis<br>Post-laminectomy kyphosis	Inclusionary
Control	M41.4<br>M00.X - M99.X<br>G80.X<br>Q90.X - Q99.X<br>E88.X	Neuromuscular scoliosis<br>Musculoskeletal diseases<br>Any CP condition<br>Range of chromosomal abnormalities<br>Metabolic disorders	Exclusionary
Cerebral Palsy	G80.0-G80.2	Spastic cerebral palsy	Inclusionary
Cerebral Palsy	G80.3<br>G80.4<br>Q90.X - Q99.X<br>E88.X	Athetoid cerebral palsy<br>Ataxic cerebral palsy<br>Range of chromosomal abnormalities<br>Metabolic disorders	Exclusionary
Epilepsy	G40.0XX<br>G40.1XX<br><br>G40.2XX	Localization-related idiopathic epilepsy<br>Localization-related symptomatic epilepsy with simple partial seizures<br>Localization-related symptomatic epilepsy with complex partial seizures	Inclusionary
Epilepsy	G40.5XX<br><br>G40.8XX<br>G40.AXX	Epileptic seizures not related to external causes<br>Other epilepsy<br>Absence epileptic syndrome	Exclusionary

Section 3

Probe-reduction by filtering. This filtering step is performed to assure that confounding variance is minimized, by removal of known high-variance probes. After filtering, 806,777 probes remained for subsequent analysis

**Procedure**	**Number of Remaining Probes**
Start	1,051,943
Remove single-nucleotide polymorphisms(SNPs)	835,424
Remove low detection-quality probes	825,328
Remove sex-chromosome probes	806,798
Removes probes with infinite M-values	806,777

Section 4

Distributions of probe performance after first iteration of bootstrapping. The scale of the graphs indicate a high number of low-performing probes and a very small number of high-performing probes.

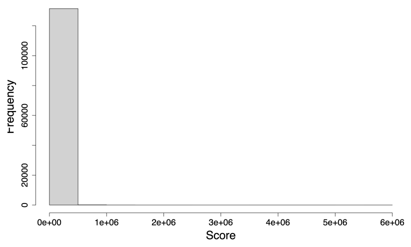

Figure 5: 4-Way SVM scores

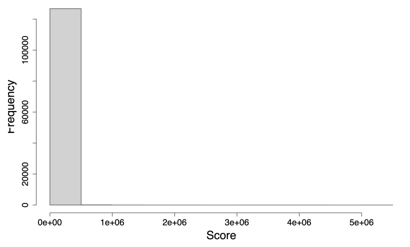

Figure 6: 4-way LDA scores

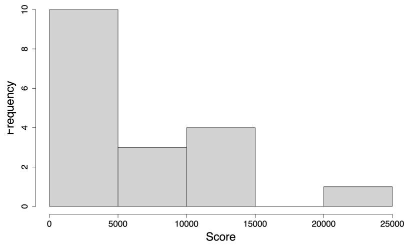

Figure 7: Binary SVM scores

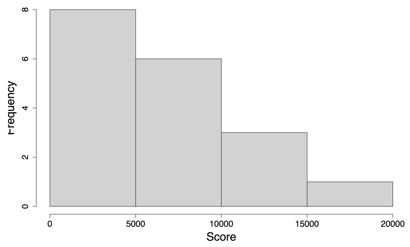

Figure 8: Binary LDA scores

Section 5

Flow Chart Representing Algorithm

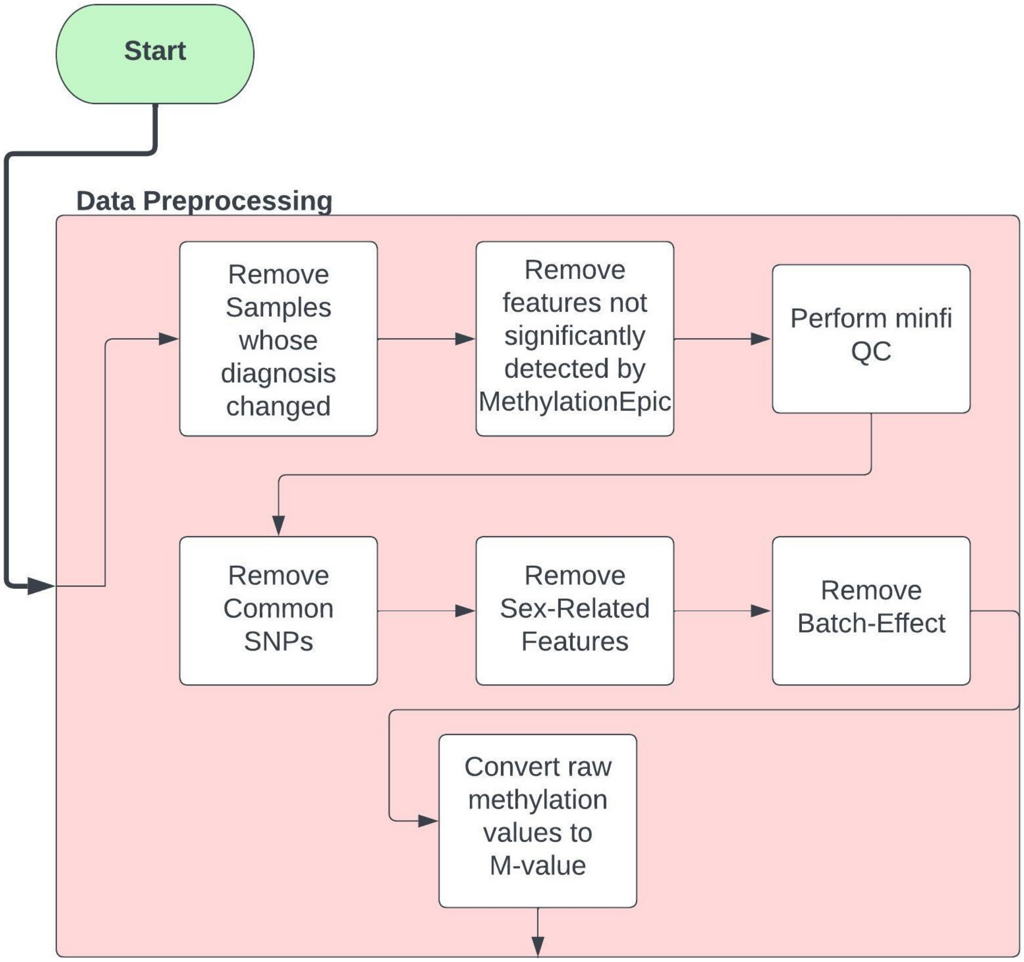

Figure 9: Data preprocessing algorithm. In the red block, data are filtered and processed from raw methylation data into M values.

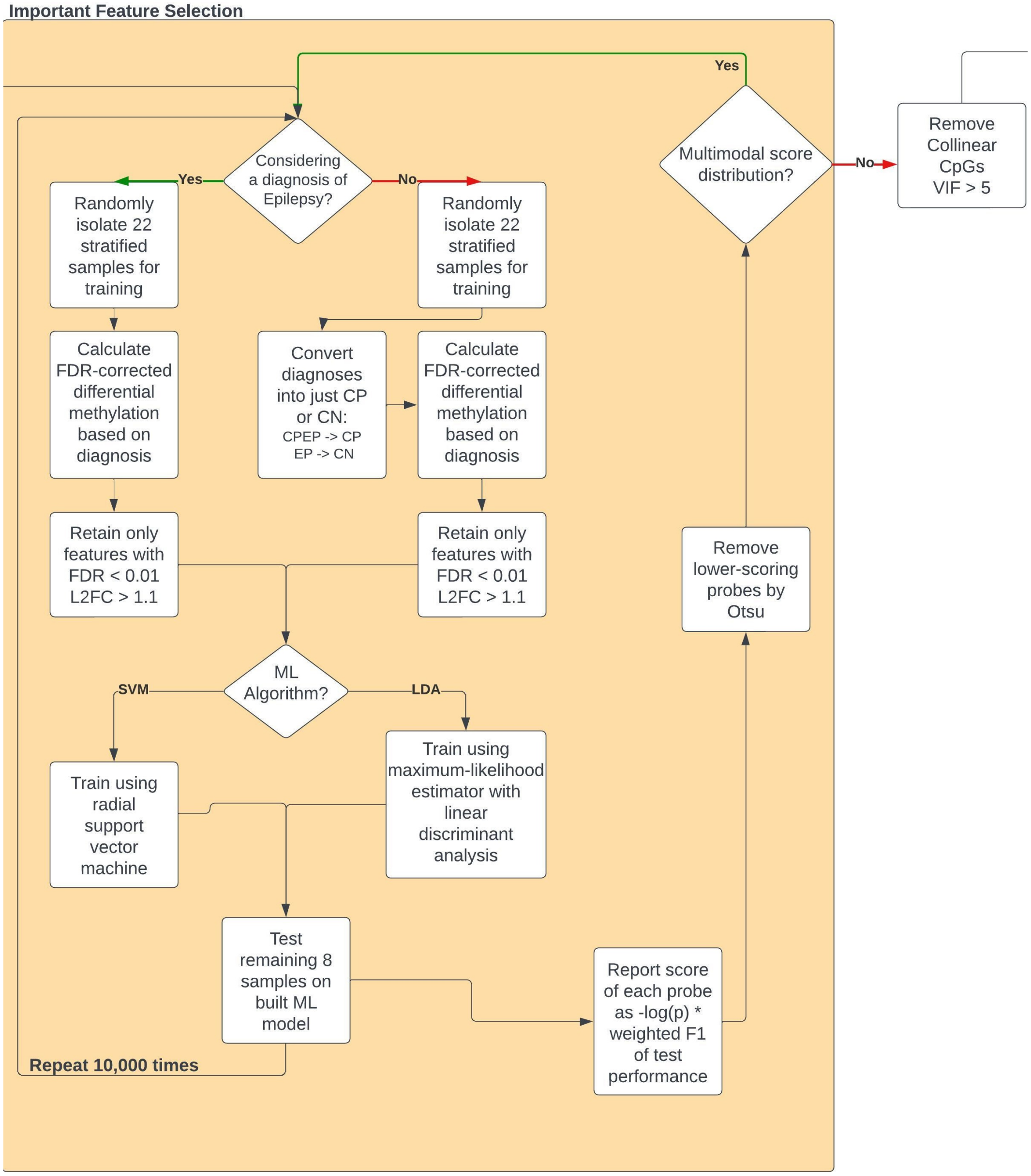

Figure 10: CpG identification algorithm.In the orange block, CpG loci are identified that have a statistical difference between groups and high capacity to classify groups.

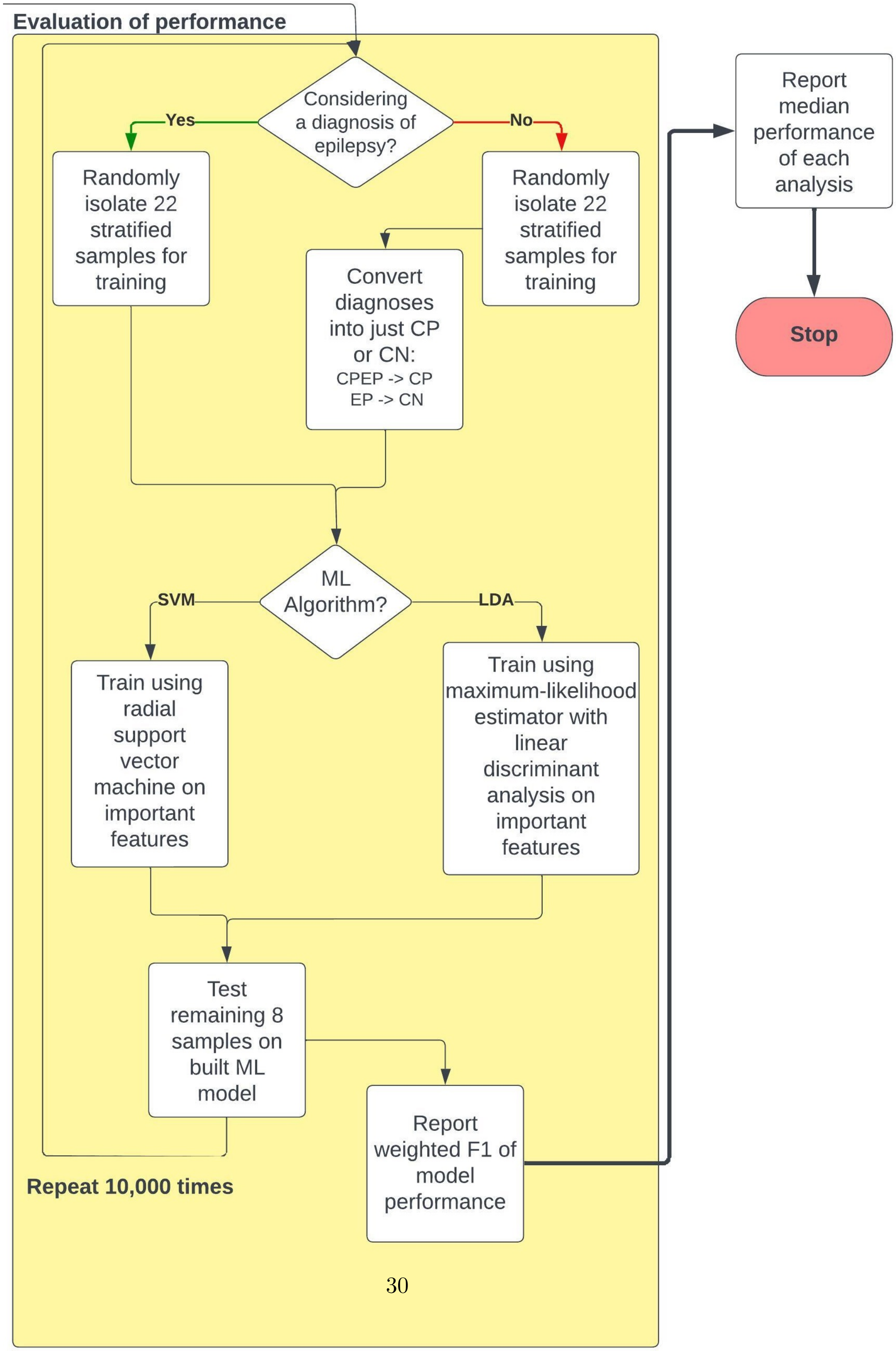

Figure 11: Classification algorithm. In the yellow block, CpG loci that have been identified are used to assess diagnostic capability in the samples.
